# Bon-EV: an improved multiple testing procedure for controlling false discovery rates

**DOI:** 10.1186/s12859-016-1414-x

**Published:** 2017-01-03

**Authors:** Dongmei Li, Zidian Xie, Martin Zand, Thomas Fogg, Timothy Dye

**Affiliations:** 1 Clinical and Translational Science Institute, School of Medicine and Dentistry, University of Rochester, 265 Crittenden Boulevard CU 420708, Rochester, 14642 NY USA; 2 Goergen Institute for Data Science, University of Rochester, Computer Studies Building, Rochester, 14642 NY USA; 3 Department of Obstetrics and Gynecology, University of Rochester, 500 Red Creek Drive Suite 220, Rochester, 14623 NY USA

**Keywords:** Multiple testing procedure, FDR, Power, Stability, RNA-Seq

## Abstract

**Background:**

Stability of multiple testing procedures, defined as the standard deviation of total number of discoveries, can be used as an indicator of variability of multiple testing procedures. Improving stability of multiple testing procedures can help to increase the consistency of findings from replicated experiments. Benjamini-Hochberg’s and Storey’s *q*-value procedures are two commonly used multiple testing procedures for controlling false discoveries in genomic studies. Storey’s *q*-value procedure has higher power and lower stability than Benjamini-Hochberg’s procedure. To improve upon the stability of Storey’s *q*-value procedure and maintain its high power in genomic data analysis, we propose a new multiple testing procedure, named Bon-EV, to control false discovery rate (FDR) based on Bonferroni’s approach.

**Results:**

Simulation studies show that our proposed Bon-EV procedure can maintain the high power of the Storey’s *q*-value procedure and also result in better FDR control and higher stability than Storey’s *q*-value procedure for samples of large size(30 in each group) and medium size (15 in each group) for either independent, somewhat correlated, or highly correlated test statistics. When sample size is small (5 in each group), our proposed Bon-EV procedure has performance between the Benjamini-Hochberg procedure and the Storey’s *q*-value procedure. Examples using RNA-Seq data show that the Bon-EV procedure has higher stability than the Storey’s *q*-value procedure while maintaining equivalent power, and higher power than the Benjamini-Hochberg’s procedure.

**Conclusions:**

For medium or large sample sizes, the Bon-EV procedure has improved FDR control and stability compared with the Storey’s *q*-value procedure and improved power compared with the Benjamini-Hochberg procedure. The Bon-EV multiple testing procedure is available as the BonEV package in R for download at https://CRAN.R-project.org/package=BonEV.

**Electronic supplementary material:**

The online version of this article (doi:10.1186/s12859-016-1414-x) contains supplementary material, which is available to authorized users.

## Background

Microarray and next-generation sequencing (NGS) technologies have been widely used in biological and biomedical research to identify novel biomarkers and genomic modifications related to biological processes and diseases. Multiple testing procedures are widely used in microarray and NGS studies to control the multiple testing error rate to minimize false discoveries from the enormous number of simultaneous hypothesis tests [1]. Many multiple testing error rates have been proposed such as family-wise error rate (FWER) [2], *k* family-wise error rate (kFWER) [3], and false discovery rate (FDR) [4]. For discovery purposes, the false discovery rate (FDR), defined as the expected proportion of false discoveries among total number of discoveries, is often controlled in multiple testing procedures to select significant features in microarray and NGS studies [4–6]. Benjamini and Hochberg’s FDR controlling procedure [4] and Storey’s *q*-value procedure [7, 8] are the most commonly used procedures [9]. The Bonferroni procedure, although perceived as a conservative procedure for multiple testing error rate control, has stability superior to Benjamini-Hochberg’s procedure in terms of variability of total number of discoveries, and equivalent power to Benjamini-Hochberg’s procedure, when used to control the expected number of false discoveries (EV, where *V* stands for the number of false discoveries) at a user-specified level [10].

In this study, we examine the stability (defined as standard deviation of the total number of rejected hypotheses) of both Benjamini-Hochberg’s FDR controlling procedure and Storey’s *q*-value procedure for generating adjusted *p*-values to select significant genes or biomarkers in microarray and NGS data analysis. In addition, we propose our own multiple testing procedure (named Bon-EV) based on Bonferroni’s EV controlling procedure, that has equivalent power, higher stability, and better FDR control than the Storey’s *q*-value procedure with at least medium-sized samples in microarray and NGS data analysis. Multiple testing procedures with high power, good FDR control, and high stability are desirable in genomic data analysis due to the high cost of sequencing in genomic studies. The Bon-EV multiple testing procedure will be attractive to genomic data analysts as it not only maintains the high power of Storey’s *q*-value procedure, but also offers better FDR control and higher stability, especially for small to medium sample size studies that need high stability, high power and good FDR control to maximize the odds of true discoveries.

## Methods

Suppose we are testing *m* null hypotheses simultaneously in a high-dimensional data analysis for single nucleotide polymorphism (SNP) identification, differential gene expression, or DNA methylation discovery. Among *m* null hypotheses, *m*_0_ null hypotheses are true null hypotheses. Among *R* rejected null hypotheses, *V* hypotheses are false rejections (false discoveries). Multiple testing error rates need to be controlled to minimize false discoveries among total rejections. False discovery rate (FDR) is a commonly used multiple testing error rate in genomic analysis. Several definitions of FDR have been proposed to measure the false discovery rate such as FDR, positive false discovery rate (pFDR), and $\frac {E(V)}{E(R)}$. The FDR and pFDR are defined as:

1 $$ FDR = E\left(\frac{V}{R}|R > 0\right)Pr(R > 0),  $$

2 $$ pFDR = E\left(\frac{V}{R}|R > 0\right).  $$

### Benjamini-Hochberg procedure

The Benjamini-Hochberg procedure [4] provides control of FDR at level *α* through the following step-up procedure:

- Order original *p*-values *p*_*i*_,*i*=1,…,*m*, from the smallest to the largest such that *p*_(1)_≤*p*_(2)_·≤*p*_(*m*)_;
- Find *k* as the largest *i* for which $P_{(i)} \le \frac {i}{m}\alpha $;
- Reject all null hypotheses *H*_*i*_,*i*=1,2,…,*k*.

The Benjamini-Hochberg procedure provides $FDR = \frac {m_{0}}{m}\alpha \le \alpha $, a strong control for FDR at level *α* for independent and positively correlated test statistics. Meanwhile, the Benjamini-Hochberg procedure is also conservative by a factor of $\frac {m_{0}}{m}$ for controlling FDR at level *α*.

### Storey’s *q*-value procedure

Arguing that where *m*_0_=*m*, one would not be interested in cases where no test is significant (*F* *D* *R*=1 in this situation), Storey [7] proposes the definition of positive false discovery rate (pFDR) that is conditional on at least one rejection. The Storey’s *q*-value procedure used for controlling pFDR improves power over the Benjamini-Hochberg procedure by including the estimation of $\pi _{0} = \frac {m_{0}}{m}$. Storey’s *q*-value procedure proceeds as follows:

- Order original *p*-values *p*_*i*_,*i*=1,…,*m*, from the smallest to the largest such that *p*_(1)_≤*p*_(2)_·≤*p*_(*m*)_;
- Find *k* as the largest *i* for which $P_{(i)} \le \frac {i}{m \hat {\pi _{0}}}\alpha $ where $\pi _{0} = \frac {m_{0}}{m}$;
- Reject all null hypothesis *H*_*i*_,*i*=1,2,…,*k*.

Storey proposes to estimate *π*_0_ conservatively by $\hat {\pi _{0}} = \frac {\sharp \{p_{i} > \lambda \}}{(1-\lambda)m}$, where *λ* is chosen to minimize the mean-squared error of the *pFDR* estimates.

### Bonferroni procedure

The Bonferroni procedure has traditionally been considered too conservative for genomic data analysis for discovery purposes. Gordon et al. [10] show the Bonferroni procedure has comparable power and superior stability to the Benjamini-Hochberg procedure when used to control the expected number of false discoveries (*E*(*V*)). The Bonferroni procedure rejects *H*_*i*_ if $p_{i} \le \frac {\gamma }{m}$, and controls *E*(*V*) at a pre-specified number of tests *γ* when test statistics are either independent or correlated. To prove that the Bonferroni procedure controls *E*(*V*) at level *γ*, we assume *p*_*i*_ for *i*=1,2,…,*m*_0_ has an independent uniform distribution, then we have

3 $$ E(V) = \sum_{i = 1}^{m_{0}}Pr\left(p_{i} \le \frac{\gamma}{m}\right) = m_{0} \frac{\gamma}{m} \le \gamma.  $$

If we let *γ*=*α*·*E*(*R*), then we have *E*(*V*)≤*α*·*E*(*R*) and $\frac {E(V)}{E(R)} \le \alpha $. Notice that the Bonferroni procedure used to control *E*(*V*) is conservative by a factor of $\frac {m_{0}}{m}$. Thus, we further improve power of the Bonferroni procedure by estimating *m*_0_ and replacing *m* with a conservative estimator of *m*_0_ in the cutoff value.

### Bon-EV procedure

Based on theorem 1 in Storey’s 2003 paper, we propose our own Bon-EV procedure to control the *pFDR* at level *α*. Theorem 1 states that: Suppose that *m* identical hypothesis tests are performed with *p*-values *P*_1_,…,*P*_*m*_ and significant region includes all adjusted *p*-values *P* ^∗^ less than or equal to *α*. Assume that (*P*_*i*_,*H*_*i*_) are *i*.*i*.*d*. random variables, *P*_*i*_|*H*_*i*_∼(1−*H*_*i*_)·*F*_0_+*H*_*i*_·*F*_1_ for some null distribution *F*_0_ and alternative distribution *F*_1_, and *H*_*i*_∼*B* *e* *r* *n* *o* *u* *l* *l* *i*(*π*_1_) for *i*=1,…,*m*. Then

4 $$ pFDR = Pr(H = 0| P^{*} \le \alpha)  $$

Based on *p* *F* *D* *R*=*P* *r*(*H*=0|*P* ^∗^≤*α*), our Bon-EV procedure is as follows:

- Compare each *p*_*i*_ with $\frac {\alpha \cdot Pr(\widehat {P^{*}} \le \alpha)}{\hat {\pi _{0}}}$, *i*=1,2,…,*m*;
- Reject *H*_*i*_ if $p_{i} \leq \frac {\alpha \cdot Pr(\widehat {P^{*}} \le \alpha)}{\hat {\pi _{0}}}$, *i*=1,2,…,*m*.

In our Bon-EV procedure, *P* ^∗^ are the adjusted *p*-values from the Benjamini-Hochberg procedure, and *P* *r*(*P* ^∗^≤*α*) is estimated by the proportion of null hypotheses that have the Benjamini-Hochberg adjusted *p*-values ≤*α*. We use the same estimate of *π*_0_ as used in Storey’s *q*-value procedure.

The following equations show that our Bon-EV procedure controls *pFDR* at level *α*.

5 $$ V = \sum_{i = 1}^{m}I(P^{*} \le \alpha \, and \, H_{i} = 0).  $$

6 $$\begin{array}{@{}rcl@{}} E(V) &=& {\sum\nolimits}_{i = 1}^{m}Pr(P^{*}_{i} \le \alpha \, and \quad H_{i} = 0) \notag \\ &=& {\sum\nolimits}_{i = 1}^{m}Pr(H_{i} = 0 | P^{*}_{i} \le \alpha)Pr(P^{*}_{i} \le \alpha) \notag \\ &=& m Pr(H = 0 | P^{*} \le \alpha)Pr(P^{*} \le \alpha) \notag \\ &=& pFDR \cdot (m Pr(P^{*} \le \alpha)). \end{array} $$

So, if *E*(*V*)≤*α*·(*m* *P* *r*(*P* ^∗^≤*α*)), then *p* *F* *D* *R*≤*α*. To have *E*(*V*)≤*α*·(*m* *P* *r*(*P* ^∗^≤*α*)) using the Bonferroni approach, we compare each *p*_*i*_ with $\frac {\alpha \cdot (m Pr(P^{*} \le \alpha))}{m_{0}}$:

7 $$\begin{array}{@{}rcl@{}} E(V) &= \sum_{i = 1}^{m_{0}}Pr\left(p_{i} \leq \frac{\alpha \cdot (m Pr(P^{*} \le \alpha))}{m_{0}}\right) \notag \\ &= \sum_{i = 1}^{m_{0}}Pr\left(p_{i} \leq \frac{\alpha \cdot (m Pr(P^{*} \le \alpha))}{m \cdot \pi_{0}}\right) \notag \\ &= m_{0} \cdot \frac{\alpha \cdot (m Pr(P^{*} \le \alpha))}{m_{0}} \notag \\ &= \alpha \cdot (m Pr(P^{*} \le \alpha)). \end{array} $$

Thus, *pFDR* will be controlled at level *α* if each *p*_*i*_ is compared with $\frac {\alpha \cdot (m Pr(P^{*} \le \alpha))}{m_{0}}$. As *m*_0_=*m*·*π*_0_, we compare each *p*_*i*_ with $\frac {\alpha \cdot Pr(P^{*} \le \alpha)}{\pi _{0}}$. *P* *r*(*P* ^∗^≤*α*) is estimated by $Pr(\widehat {P^{*}} \le \alpha) = \frac {\sharp \{P^{*}_{i} \le \alpha \}}{m}$ from Benjamini-Hochberg’s FDR controlling method and *π*_0_ is estimated by $\hat {\pi _{0}} = \frac {\sharp \{p_{i} > \lambda \}}{(1-\lambda)m}$ from Storey’s *q*-value method. The expected values of $Pr(\widehat {P^{*}} \le \alpha)$ and $\hat {\pi _{0}}$ are

8 $$\begin{array}{@{}rcl@{}} E(Pr(\widehat{P^{*}} \le \alpha)) &= E\left[\frac{\sharp\{P^{*}_{i} \le \alpha\}}{m}\right] \notag \\ &= Pr(P^{*} \le \alpha); \end{array} $$

9 $$ E(\hat{\pi_{0}}) = E\left[\frac{\sharp\{p_{i} > \lambda\}}{(1-\lambda)m}\right] =\frac{(1-\lambda)m}{(1-\lambda)m} = 1 \geq \pi_{0}.  $$

Thus, our procedure controls *E*(*V*) at *α*·(*m* *P* *r*(*P* ^∗^≤*α*)) and controls *pFDR* at level *α*. We took advantage of existing R functions to estimate *P* *r*(*P* ^∗^≤*α*) and *π*_0_. We estimate *P* *r*(*P* ^∗^≤*α*) from the *p.adjust* function in R with Benjamini and Hochberg’s method and estimate *π*_0_ using the *qvalue* and *pi0est* function from the *qvalue* package.

Our proposed Bon-EV procedure integrates the approaches of the Bonferroni procedure, Benjamini-Hochberg procedure, and Storey’s *q*-value procedure. The estimated *π*_0_ from Storey’s *q*-value procedure used in the Bon-EV procedure increases the cutoff value for *p*-values in each comparison, thus improving its power compared to Benjamini-Hochberg’s procedure. As the sample size increases, the $\hat {\pi _{0}}$ is closer to the value of *π*_0_, and the power will be further improved. By adapting the estimate of *P* *r*(*P* ^∗^≤*α*) from the Benjamini-Hochberg procedure for the Bon-EV procedure, the proportion of false discoveries is reduced compared to Storey’s *q*-value procedure. Thus, we expect the Bon-EV procedure to have better FDR control than Storey’s *q*-value procedure. Regarding the stability, single-step approaches such as the Bonferroni and Bon-EV procedures are superior to stepwise approaches such as the Benjamini-Hochberg procedure and Storey’s *q*-value procedure, but, the inclusion of the *π*_0_ estimate in the Bon-EV and Storey’s *q*-value procedures reduces the stability. Taken together, we expect the Bon-EV procedure to have better stability than Storey’s *q*-value procedure.

## Results

### Simulation studies

We conduct simulation studies to evaluate the FDR control, power, and stability of our Bon-EV procedure, the Benjamini-Hochberg procedure and Storey’s *q*-value procedure. Power is defined as the proportion of true rejections among total non-true null hypotheses, and stability is defined as the standard deviation (SD) of total number of rejections [11].

10 $$ Power = E\left(\frac{S}{m - m_{0}}\right),  $$

11 $$ Stability = SD(R).  $$

Our simulations compare gene expression levels between two groups with equal sample sizes of 5, 15, and 30 in each group. For each sample, we test 10,000 genes with expression levels following multivariate normal distributions with means at vector of 0 for the control group and means at vector of (*μ*,0) for the treatment group. We set standard deviations at 1 with correlations between genes equal to *ρ* (*ρ* = 0, 0.4, or 0.8, depending on the simulation study). All genes were equally correlated with correlation *ρ*. Meanwhile, we also conduct simulations with pairwise gene correlations randomly selected from uniform(0, 0.8) distribution for sample sizes of 5, 15, and 30 in each of the two groups. We set the proportions of differentially expressed genes (*π*_1_=1−*π*_0_) at 0.005, 0.01, 0.05, 0.10, 0.15, 0.20, 0.25, 0.30, 0.40, 0.50, 0.60, 0.75, and 0.80 for each simulation study. The vector *μ* is set as a sequence from 1 to 3 with length equal to 10,000×*π*_1_. We use *t*-statistics for two independent samples for testing differential gene expression between groups. Each simulation include 1,000 iterations. Each procedure is set to control the FDR at the 0.05 level.

### Simulation results

**Fig. 1:**
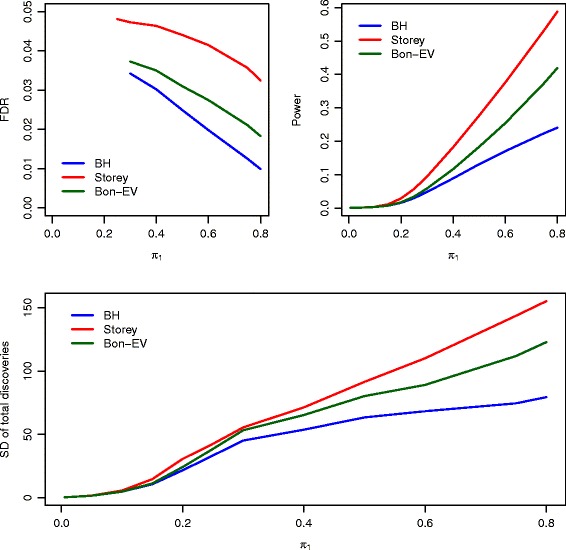
Estimated FDR, power, SD of power, and SD of total number of discoveries of compared multiple testing procedures with *ρ*=0 and sample size of 5 in each group. *Blue*: Benjamini-Hochberg procedure; *Red*: Storey’s *q*-value procedure; *Dark Green*: Bon-EV procedure

Figure 1 illustrates FDR, power, and stability estimates of the three multiple testing procedures for small sample size (5 in each group) when *ρ*=0. For test statistics that were independent from each other (*ρ*=0),the Benjamini-Hochberg procedure has the strongest FDR control (the smallest FDR), followed by the Bon-EV procedure. Storey’s *q*-value procedure has the largest FDR, although all three procedures control FDR within 5%. It is also noticeable that the estimated FDR decreases as the proportion of non-true null hypotheses increase for all three multiple testing procedures. Storey’s *q*-value procedure has the greatest power, followed first by the Bon-EV procedure, and then the Benjamini-Hochberg procedure (Additional file 1: Table S1). All three multiple testing procedures have very low power when the proportions of non-true null hypotheses are less than 15%, and the power of all multiple testing procedures increases as proportions of non-true null hypotheses increase. The Benjamini-Hochberg procedure is the most stable, followed by the Bon-EV procedure, with Storey’s *q*-value procedure the least stable. The Benjamini-Hochberg procedure has the smallest SD of total number of discoveries and Storey’s *q*-value procedure has the largest SD of total number of discoveries (Additional file 1: Figure S1). When test statistics are moderately correlated (*ρ*=0.4), the same trends are observed for FDR, power, and SD of total number of discoveries. We notice the power of our Bon-EV procedure and Storey’s *q*-value procedure converge as the correlation between test statistics increases from 0 to 0.4. Meanwhile, the power of all three procedures increases as correlations of test statistics increase especially when the proportions of non-true null hypotheses are small. When correlations within test statistics further increase to 0.8, we observe the same trends for FDR, power, and SD of total number of discoveries (Additional file 1: Figure S2). It is also noticeable that the difference in power between Storey’s *q*-value procedure and our Bon-EV procedure gets increasingly smaller as test statistic correlations increase to 0.8 from 0.4. The total number of discoveries (Additional file 1: Table S3) increase as the correlation of test statistics increases. The total number of discoveries of our Bon-EV procedure is close to Storey’s *q*-value procedure and higher than the Benjamini-Hochberg procedure. In these small sample size cases, a large proportion of non-true null hypotheses are not detected, especially when the proportion of non-true null hypotheses is small. The FDR, power, and stability estimates when the correlations across genes are random are close to the estimates when correlations across genes are 0.4 (*ρ*=0.4, Additional file 1: Figure S3 and Additional file 1: Table S1).

**Fig. 2:**
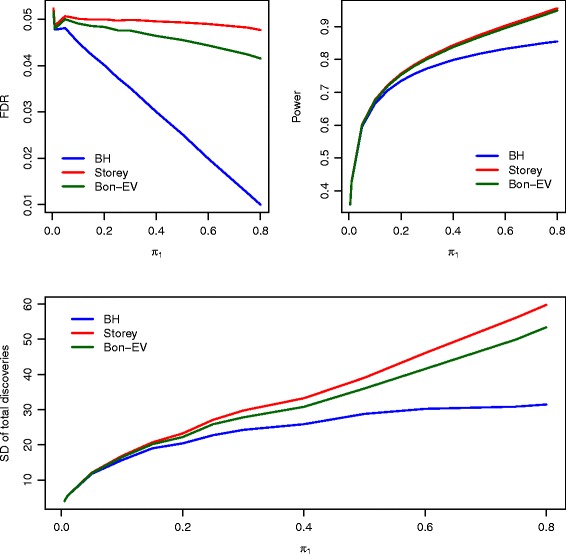
Estimated FDR, power, SD of power, and SD of total number of discoveries of compared multiple testing procedures with *ρ*=0 and sample size of 15 in each group. *Blue*: Benjamini-Hochberg procedure; *Red*: Storey’s *q*-value procedure; *Dark Green*: Bon-EV procedure

Figure 2 illustrates the FDR, power, and SD of total number of discoveries of the three multiple testing procedures at sample sizes of 15 in each group when *ρ*=0. At these sample sizes and with independent test statistics (*ρ*=0), all three multiple testing procedures control FDR to within 5% except when the proportion of non-true null hypotheses is very small. The power of our Bon-EV procedure is very close to Storey’s *q*-value procedure and higher than the Benjamini-Hochberg procedure when the proportion of non-true null hypotheses is greater than 0.15 (Additional file 1: Table S2). Storey’s *q*-value procedure has the lowest stability (the highest SD of total number of discoveries) among the three multiple testing procedures especially when the proportion of non-true null hypotheses is large. We observe the same trend at a moderate level of correlation between test statistics (*ρ*=0.4). The power of our Bon-EV procedure is the same as the power of Storey’s *q*-value procedure, and the Bon-EV procedure continues to show greater stability (smaller SD of total number of discoveries) than Storey’s *q*-value procedure (Additional file 1: Figure S4). At the highest level of correlation between test statistics (*ρ*=0.8), the trends remain the same (Additional file 1: Figure S5). The power of our Bon-EV procedure and Storey’s *q*-value procedure is greater than the Benjamini-Hochberg procedure, and the Benjamini-Hochberg procedure has better FDR control and lower SD of total number of discoveries. Our Bon-EV procedure still has better FDR control, the same power, and higher stability than Storey’s *q*-value procedure. The total number of discoveries is much closer to the true number of non-true null hypotheses when the sample size increases to 15 in each group (Additional file 1: Table S3). The total number of discoveries for the Bon-EV procedure is almost the same as for Storey’s *q*-value procedure, and still higher than the Benjamini-Hochberg procedure. Also, for Storey’s *q*-value procedure, the total number of discoveries exceeds the number of non-true null hypotheses when the proportion of non-true null hypotheses is larger than 40%. Similar results on FDR, power, and stability estimates are observed when the data have random correlations (Additional file 1: Figure S6 and Additional file 1: Table S2).

**Fig. 3:**
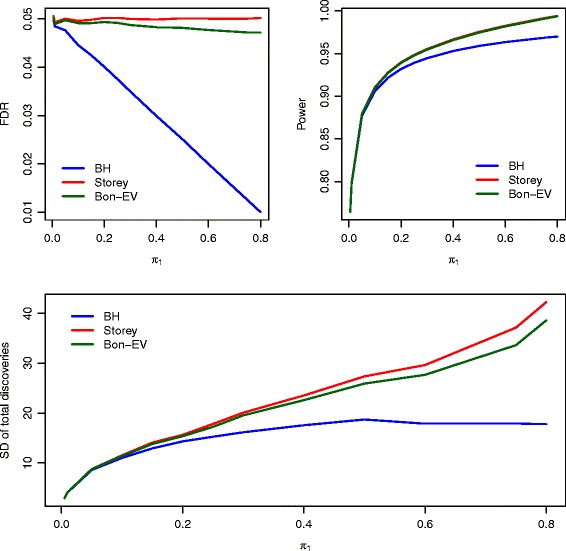
Estimated FDR, power, SD of power, and SD of total number of discoveries of compared multiple testing procedures with *ρ*=0 and sample size of 30 in each group. *Blue*: Benjamini-Hochberg procedure; *Red*: Storey’s *q*-value procedure; *Dark Green*: Bon-EV procedure

Figure 3 shows the FDR, power, and SD of total number of discoveries of the three multiple testing procedures when *ρ*=0 and sample size is as large as 30 in each group. All three multiple testing procedures illustrate reasonable control of FDR to within 5%. The power of our Bon-EV procedure is equivalent to Storey’s *q*-value procedure but higher than the Benjamini-Hochberg procedure when the proportion of non-true null hypotheses is greater than 0.15 (Additional file 1: Table S3). Storey’s *q*-value procedure still has the lowest stability among the three multiple testing procedures especially when the proportion of non-true null hypotheses is large. We observe similar results on FDR, power, and stability estimates at a moderate and high level of correlations between test statistics when *ρ*=0.4 and *ρ*=0.8 (Additional file 1: Figure S7 and Additional file 1: Figure S8). We also notice that the power and stability improve as the correlation and sample size increase. The FDR, power, and stability estimates when data have random correlations are similar to those estimates when data has moderate correlations (*ρ*=0.4, Additional file 1: Figure S9 and Additional file 1: Table S3 and Table S4).

### Examples using real data

As a complement to our simulation studies, we also compare apparent test power (total number of discoveries) and stability (SD of total number of discoveries) resulting from these three different procedures using human RNA-Seq data [12]. We compare gene expression levels between 17 females and 24 males using count data from RNA-Seq downloaded from the ReCount web site [13]. The RNA-Seq data from human B-cells that we analyze include 52,580 genes and 41 samples. The summarized count data from the RNA-Seq experiment is first filtered by only retaining genes that express at a count-per-million (CPM) above 0.5 in at least two samples. The retained 9745 genes are further normalized to eliminate RNA composition biases between libraries by finding a set of scaling factors for the library sizes that minimize the log-fold changes between the samples for most genes using the default method-a trimmed mean of M values (TMM) [14] between each pair of samples. The raw *p*-value is obtained by fitting negative binomial generalized linear models with Cox-Reid dispersion estimates using the glmFit and flmLRT function in the edgeR package in Bioconductor [15].

**Fig. 4:**
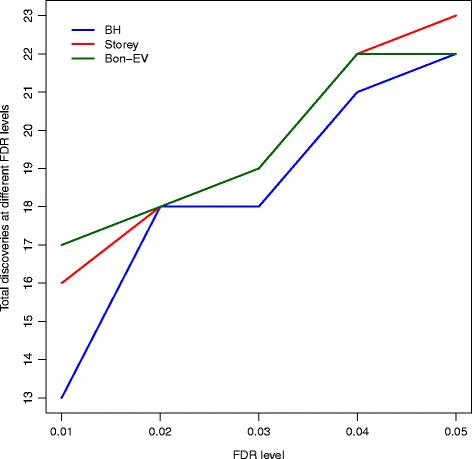
Total number of discoveries from human RNA-Seq data by three different multiple testing procedures from Cheung data. *Blue*: Benjamini-Hochberg procedure; *Red*: Storey’s *q*-value procedure; *Dark Green*: Bon-EV procedure

We apply the three multiple testing procedures to the raw *p*-values generated from the edgeR package to compare the total number of rejected genes (apparent test power) after controlling the false discovery rate. Figure 4 shows the apparent test powers of these three different multiple testing procedures at different FDR levels ranging from 0.01 to 0.05. The Bon-EV procedure and Storey’s *q*-value procedure produce the same number of rejections at the FDR level of 0.02 to 0.04. The Bon-EV procedure discovers more genes than the Benjamini-Hochberg procedure and Storey’s *q*-value procedure when the FDR level is equal to 1%. The apparent test power comparison is consistent with our simulation results.

**Fig. 5:**
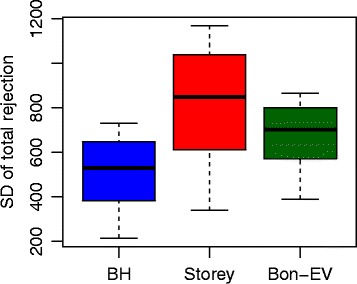
Stability comparisons among three different multiple testing procedures using Cheung data. *Blue*: Benjamini-Hochberg procedure; *Red*: Storey’s *q*-value procedure; *Dark Green*: Bon-EV procedure

To examine the stability of the Bon-EV procedure, we bootstrap the RNA-Seq samples 1000 times within each group and obtain total number of rejections in each boostrap sample. Then, we examine the stability of the Bon-EV, the Benjamini-Hochberg procedure and the Storey’s *q*-value procedure by calculating the standard deviation of total number of rejections from 1000 bootstrap samples. Figure 5 shows the stability comparison results among these three procedures, which are also consistent with the results from our simulation studies.

**Fig. 6:**
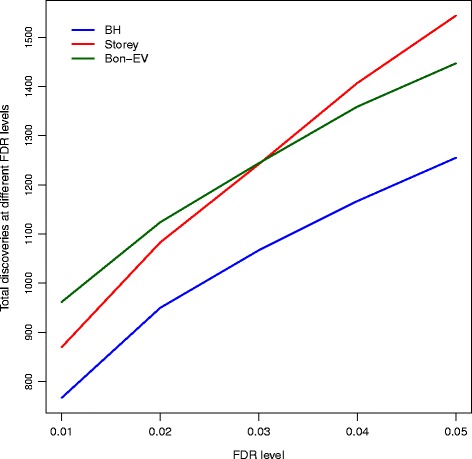
Total number of discoveries from human RNA-Seq data by three different multiple testing procedures from Bottomly data. *Blue*: Benjamini-Hochberg procedure; *Red*: Storey’s *q*-value procedure; *Dark Green*: Bon-EV procedure

**Fig. 7:**
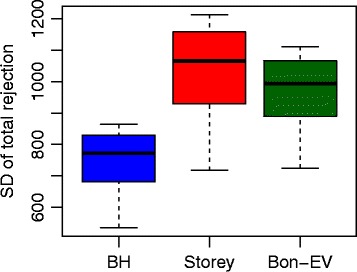
Stability comparisons among three different multiple testing procedures using Bottomly data. *Blue*: Benjamini-Hochberg procedure; *Red*: Storey’s *q*-value procedure; *Dark Green*: Bon-EV procedure

Using a similar approach, we compare apparent test power and stability of the three multiple testing procedures using *p*-values generated from a differential gene expression analysis of the two most commonly used inbred mouse strains in neuroscience research - C57BL/6J (B6) and DBA/2J (D2) [16]. Data from 10 B6 mouse samples and 11 D2 mouse samples in the RNA-Seq experiment and the count data are again downloaded from the ReCount web site. Using the same filtering criteria with *C* *P* *M*>0.5 in at least two samples, we retain 11471 genes out of 36536 total genes. After using the same analysis method from the edgeR package in Bioconductor to obtain the raw *p*-values, we apply the three different multiple testing procedures to calculate adjusted *p*-values. Figure 6 shows that the number of discoveries at different FDR levels ranges from 0.01 to 0.05. Our Bon-EV procedure also has more discoveries at lower FDR levels. The stability shown in Fig. 7 indicates that the Bon-EV procedure has higher stability than Storey’s *q*-value procedure. Again, the results are consistent with our simulation results.

## Discussion

In this study, we propose a new multiple testing procedure (Bon-EV), based on the Bonferroni procedure, intended to improve FDR control and stability as well as maintain power. We compare the Bon-EV procedure with the Benjamini-Hochberg procedure and Storey’s *q*-value procedure using both simulation studies and real RNA-Seq data. Our studies show that the proposed Bon-EV multiple testing procedure has better control of FDR and higher stability than Storey’s *q*-value procedure, and also maintains high levels of power at small and medium sample sizes.

Next generation sequencing and third generation sequencing technology has become more popular in biological and biomedical studies. The sample size in sequencing studies remain small to medium although the price of sequencing per sample has significantly decreased in recent years. Multiple testing procedures with larger power could help increase the probability of novel discoveries. The Bon-EV procedure, similar to the Storey’s *q*-value procedure, offers higher power than the Benjamini-Hochberg procedure and increases the cost-effectiveness of sequencing studies.

Compared to the Storey’s *q*-value procedure, the Bon-EV procedure offers better FDR control. In recent years, irreproducibility of results in biomedical research has drawn increasing attention in the popular press and academia [17, 18]. Investigations have found many landmarks in preclinical oncology to be non-reproducible, and confirming research conducted by scientists in the haematology and oncology department at the biotechnology firm Amgen in Thousand Oaks, California, finds only 11% of scientific findings they examined to be reproducible [17]. The reasons for irreproducibility are at least, in part, due to false discoveries in those studies [18–20]. With better FDR control, the Bon-EV procedure will enable more accurate control of false discoveries in genomic studies. Meanwhile, understanding the source of variation in the data generation and analysis can help improve reproducibility of scientific studies [21]. Multiple testing procedures are widely used to select significant features such as genes, SNPs, methylation loci, and others in microarray and NGS studies. Assessment of the stability of statistical findings from multiple testing procedures and improving the stability of these procedures could reduce replication failures. The Bon-EV procedure will help reduce replication failures compared with the Storey’s *q*-value procedure, and provide higher stability.

## Conclusions

Our study investigates the stability of Benjamini-Hochberg and Storey’s *q*-value FDR controlling procedures commonly used in genomic and genetic data analysis and proposes a new multiple testing procedure with higher stability, better FDR control and power equivalent to Storey’s *q*-value procedure as well as higher power than the Benjamini-Hochberg procedure. The Bon-EV multiple testing procedure we propose is attractive in microarray and sequencing data analysis in that it has higher power than the Benjamini-Hochberg procedure, and better FDR control and higher stability than Storey’s *q*-value procedure.

## Additional file

Additional file 1 The additional file includes supplemental figures when correlations were 0.4, 0.8, and random across all genes. Figures S1-S9 shows the estimated FDR, power, SD of power, and SD of total discoveries of compared multiple testing procedures, with correlations of 0.4, 0.8, and random, and sample size of 5, 15, and 30 in each group. The values of power and stability were shown in supplemental Table S1-S4. **Figure S1.** Shows the performance of compared multiple testing procedures with *ρ* = 0.4 and sample size of 5 in each group. **Figure S2.** Shows the performance of compared multiple testing procedures with *ρ*= 0.8 and sample size of 5 in each group. **Figure S3.** Shows the performance of compared multiple testing procedures with random correlation across genes and sample size of 5 in each group. **Figure S4.** Shows the performance of compared multiple testing procedures with *ρ* = 0.4 and sample size of 15 in each group. **Figure S5.** Shows the performance of compared multiple testing procedures with *ρ* = 0.8 and sample size of 15 in each group. **Figure S6.** Shows the performance of compared multiple testing procedures with random correlation across genes and sample size of 15 in each group. **Figure S7.** Shows the performance of compared multiple testing procedures with *ρ* = 0.4 and sample size of 30 in each group. **Figure S8.** Shows the performance of compared multiple testing procedures with *ρ* = 0.8 and sample size of 30 in each group. **Figure S9.** Shows the performance of compared multiple testing procedures with random correlation across genes and sample size of 30 in each group. **Table S1.** Shows the power and stability of compared multiple testing procedures for sample size n = 5 in each group. **Table S2.** Shows the power and stability of compared multiple testing procedures for sample size n = 15 in each group. **Table S3.** Shows the power and stability of compared multiple testing procedures for sample size n = 30 in each group. **Table S4.** Shows the total number of rejections of compared multiple testing procedures for sample size n = 5, 15, 30 in each group. (PDF 59 kb)
