# Supplementary material for: Bon-EV: an improved multiple testing procedure for controlling false discovery rates
Source: BMC Bioinformatics. 2017 Jan 3;18:1. doi: 10.1186/s12859-016-1414-x (PMC5210267; doi:10.1186/s12859-016-1414-x)
Supplement: Additional file 1 — The additional file includes supplemental figures when correlations were 0.4, 0.8, and random across all genes. Figures S1-S9 shows the estimated FDR, power, SD of power, and SD of total discoveries of compared multiple testing procedures, with correlations of 0.4, 0.8, and random, and sample size of 5, 15, and 30 in each group. The values of power and stability were shown in supplemental Table S1-S4. Figure S1. Shows the performance of compared multiple testing procedures with ρ = 0.4 and sample size of 5 in each group. Figure S2. Shows the performance of compared multiple testing procedures with ρ= 0.8 and sample size of 5 in each group. Figure S3. Shows the performance of compared multiple testing procedures with random correlation across genes and sample size of 5 in each group. Figure S4. Shows the performance of compared multiple testing procedures with ρ = 0.4 and sample size of 15 in each group. Figure S5. Shows the performance of compared multiple testing procedures with ρ = 0.8 and sample size of 15 in each group. Figure S6. Shows the performance of compared multiple testing procedures with random correlation across genes and sample size of 15 in each group. Figure S7. Shows the performance of compared multiple testing procedures with ρ = 0.4 and sample size of 30 in each group. Figure S8. Shows the performance of compared multiple testing procedures with ρ = 0.8 and sample size of 30 in each group. Figure S9. Shows the performance of compared multiple testing procedures with random correlation across genes and sample size of 30 in each group. Table S1. Shows the power and stability of compared multiple testing procedures for sample size n = 5 in each group. Table S2. Shows the power and stability of compared multiple testing procedures for sample size n = 15 in each group. Table S3. Shows the power and stability of compared multiple testing procedures for sample size n = 30 in each group. Table S4. Shows the total number of rejections of compared multiple [file 12859_2016_1414_MOESM1_ESM.pdf]

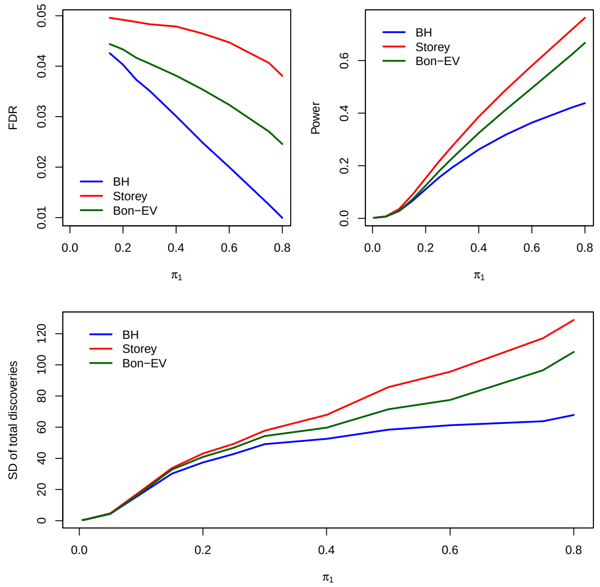

Figure 1: Estimated FDR, power, SD of power, and SD of total discoveries of compared multiple testing procedures with $\rho = 0.4$ and sample size of 5 in each group. Blue: Benjamini-Hochberg procedure; Red: Storey's q-value procedure; Dark green: Bon-EV procedure.

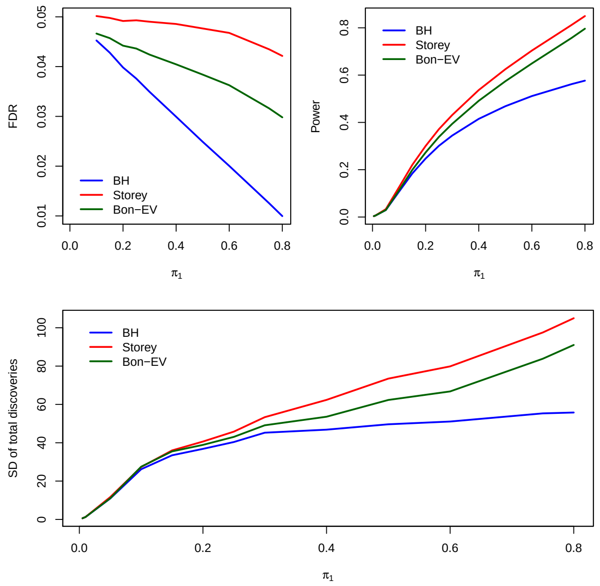

Figure 2: Estimated FDR, power, SD of power, and SD of total discoveries of compared multiple testing procedures with $\rho = 0.8$ and sample size of 5 in each group. Blue: Benjamini-Hochberg procedure; Red: Storey's q-value procedure; Dark green: Bon-EV procedure.

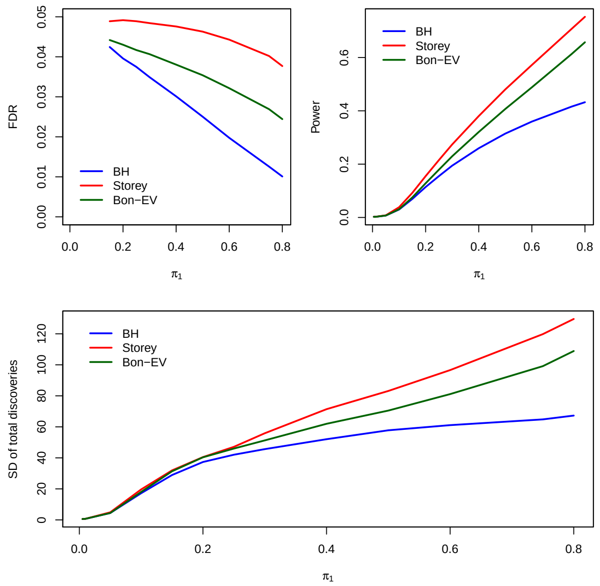

Figure 3: Estimated FDR, power, SD of power, and SD of total discoveries of compared multiple testing procedures with random correlation across genes and sample size of 5 in each group. Blue: Benjamini-Hochberg procedure; Red: Storey's q-value procedure; Dark green: Bon-EV procedure.

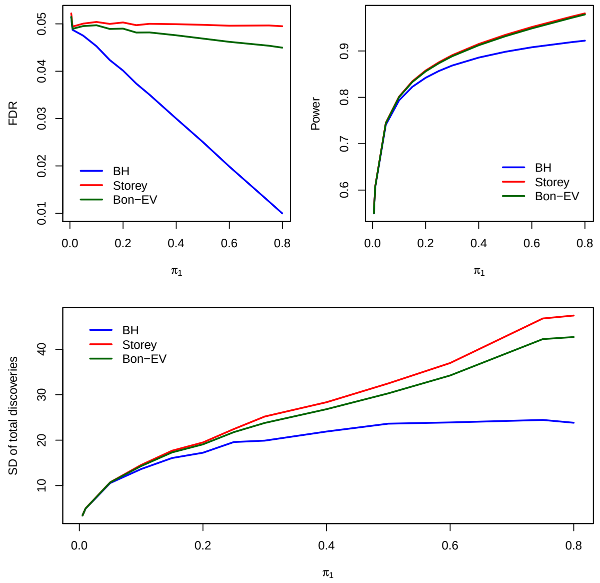

Figure 4: Estimated FDR, power, SD of power, and SD of total discoveries of compared multiple testing procedures with $\rho = 0.4$ and sample size of 15 in each group. Blue: Benjamini-Hochberg procedure; Red: Storey's q-value procedure; Dark green: Bon-EV procedure.

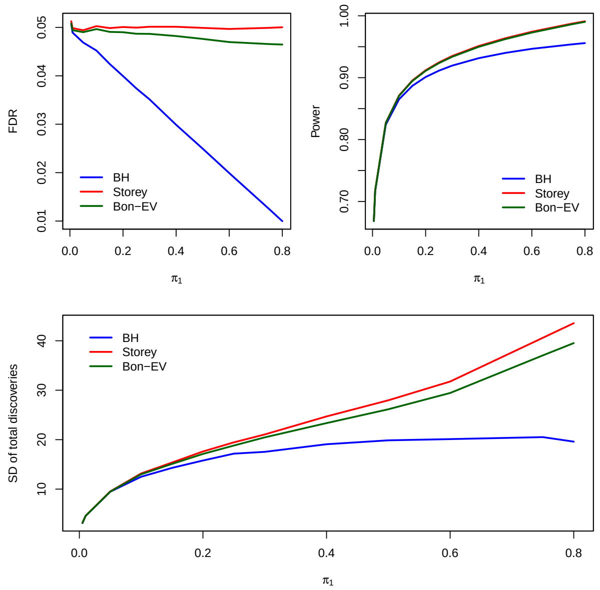

Figure 5: Estimated FDR, power, SD of power, and SD of total discoveries of compared multiple testing procedures with $\rho = 0.8$ and sample size of 15 in each group. Blue: Benjamini-Hochberg procedure; Red: Storey's q-value procedure; Dark green: Bon-EV procedure.

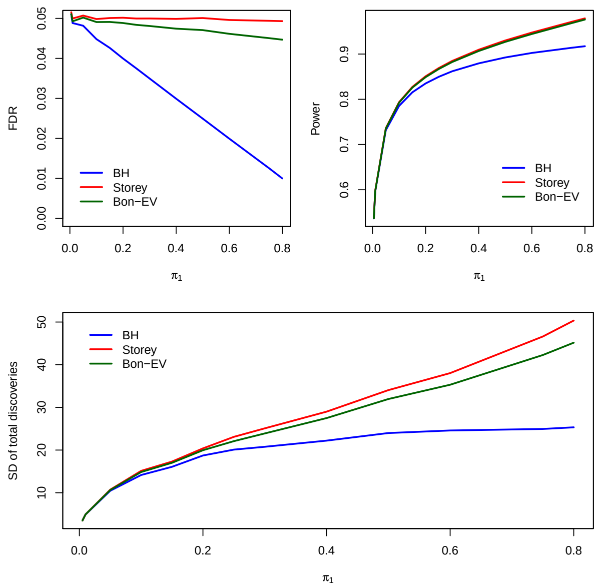

Figure 6: Estimated FDR, power, SD of power, and SD of total discoveries of compared multiple testing procedures with random correlation across genes and sample size of 15 in each group. Blue: Benjamini-Hochberg procedure; Red: Storey's q-value procedure; Dark green: Bon-EV procedure.

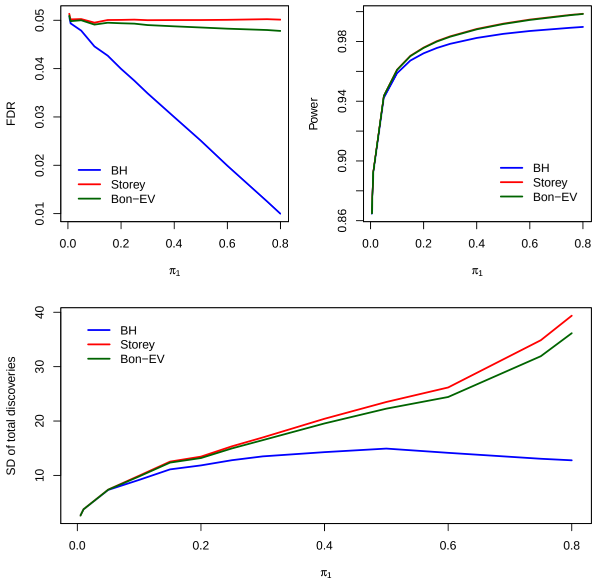

Figure 7: Estimated FDR, power, SD of power, and SD of total discoveries of compared multiple testing procedures with $\rho = 0.4$ and sample size of 30 in each group. Blue: Benjamini-Hochberg procedure; Red: Storey's q-value procedure; Dark green: Bon-EV procedure.

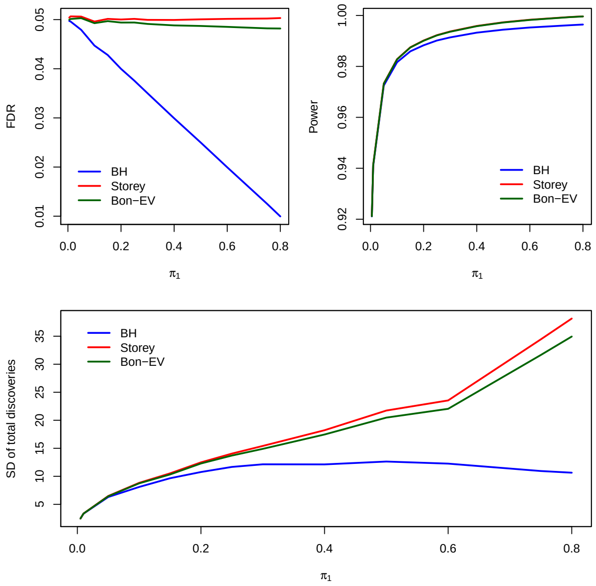

Figure 8: Estimated FDR, power, SD of power, and SD of total discoveries of compared multiple testing procedures with $\rho = 0.8$ and sample size of 30 in each group. Blue: Benjamini-Hochberg procedure; Red: Storey's q-value procedure; Dark green: Bon-EV procedure.

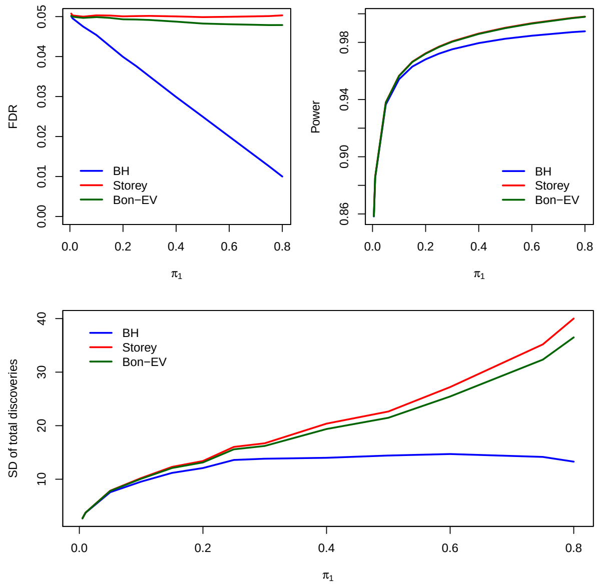

Figure 9: Estimated FDR, power, SD of power, and SD of total discoveries of compared multiple testing procedures with random correlation across genes and sample size of 30 in each group. Blue: Benjamini-Hochberg procedure; Red: Storey's q-value procedure; Dark green: Bon-EV procedure.

Table 1: Power and Stability of compared multiple testing procedures for sample size $n = 5$ in each group

ρ	π_1	Power			Stability		
		BH	Storey	Bon-EV	BH	Storey	Bon-EV
0	0.005	0.00	0.00	0.00	0.38	0.39	0.38
	0.01	0.00	0.00	0.00	0.49	0.49	0.49
	0.05	0.00	0.00	0.00	1.59	1.72	1.59
	0.10	0.00	0.00	0.00	4.82	5.75	4.89
	0.15	0.01	0.01	0.01	10.74	14.70	11.31
	0.20	0.02	0.03	0.02	21.69	30.69	24.26
	0.25	0.03	0.06	0.03	33.41	42.59	38.60
	0.30	0.05	0.09	0.06	45.22	55.57	53.33
	0.40	0.09	0.18	0.12	53.78	71.41	65.38
	0.50	0.13	0.28	0.18	63.48	91.55	80.32
	0.60	0.17	0.38	0.25	68.38	110.18	89.14
	0.75	0.22	0.53	0.37	74.60	143.76	111.96
	0.80	0.24	0.59	0.42	79.48	155.30	122.90
0.40	0.005	0.00	0.00	0.00	0.42	0.42	0.42
	0.01	0.00	0.00	0.00	0.73	0.74	0.73
	0.05	0.01	0.01	0.01	4.36	4.70	4.39
	0.10	0.03	0.04	0.03	17.30	19.13	18.07
	0.15	0.07	0.09	0.07	30.21	33.78	32.81
	0.20	0.11	0.15	0.12	37.36	43.10	40.91
	0.25	0.15	0.22	0.18	42.82	49.32	46.81
	0.30	0.19	0.27	0.23	49.10	57.75	54.31
	0.40	0.26	0.39	0.32	52.50	67.88	59.67
	0.50	0.32	0.49	0.41	58.38	85.67	71.48
	0.60	0.36	0.58	0.50	61.24	95.58	77.47
	0.75	0.42	0.72	0.62	63.79	117.04	96.50
	0.80	0.44	0.76	0.67	67.81	128.80	108.28
0.8	0.005	0.00	0.00	0.00	0.59	0.60	0.59
	0.01	0.01	0.01	0.01	1.25	1.27	1.25
	0.05	0.03	0.03	0.03	10.92	11.65	11.03
	0.10	0.11	0.13	0.11	26.18	27.35	27.50
	0.15	0.18	0.22	0.20	33.46	36.00	35.49
	0.20	0.25	0.30	0.27	36.81	40.63	38.89
	0.25	0.30	0.37	0.34	40.38	45.81	43.06
	0.30	0.34	0.43	0.39	45.25	53.34	49.12
	0.40	0.41	0.54	0.49	46.83	62.37	53.58
	0.50	0.47	0.62	0.57	49.63	73.47	62.33
	0.60	0.51	0.70	0.65	51.08	79.86	66.79
	0.75	0.56	0.81	0.76	55.34	97.55	83.85
	0.80	0.58	0.85	0.80	55.78	104.98	91.03
random	0.005	0.00	0.00	0.00	0.56	0.56	0.56
	0.01	0.00	0.00	0.00	0.71	0.71	0.71
	0.05	0.01	0.01	0.01	4.44	4.89	4.44
	0.10	0.03	0.04	0.03	17.21	19.65	18.02
	0.15	0.07	0.09	0.08	28.94	31.94	31.35
	0.20	0.11	0.15	0.13	37.34	40.50	40.36
	0.25	0.16	0.22	0.18	42.03	47.10	46.01
	0.30	0.19	0.27	0.23	45.64	55.87	51.27
	0.40	0.26	0.38	0.32	52.05	71.41	61.99
	0.50	0.31	0.48	0.41	57.79	83.16	70.51
	0.60	0.36	0.57	0.49	61.11	96.63	81.14
	0.75	0.42	0.71	0.61	64.83	119.82	99.16
	0.80	0.43	0.75	0.66	67.28	129.60	108.91

Table 2: Power and Stability of compared multiple testing procedures for sample size $n = 15$ in each group

ρ	π_1	Power			Stability		
		BH	Storey	Bon-EV	BH	Storey	Bon-EV
0	0.005	0.36	0.36	0.36	3.96	3.98	3.96
	0.01	0.43	0.43	0.43	5.49	5.50	5.51
	0.05	0.60	0.60	0.60	11.75	12.06	11.96
	0.10	0.67	0.68	0.67	15.66	16.85	16.50
	0.15	0.71	0.72	0.72	18.99	20.69	20.16
	0.20	0.73	0.76	0.75	20.41	23.23	22.21
	0.25	0.76	0.78	0.78	22.72	27.09	25.84
	0.30	0.77	0.81	0.80	24.24	29.74	27.80
	0.40	0.80	0.84	0.84	25.85	33.23	30.79
	0.50	0.82	0.87	0.87	28.79	39.06	36.01
	0.60	0.83	0.90	0.90	30.22	46.08	41.56
	0.75	0.85	0.94	0.94	30.84	56.12	49.93
	0.80	0.85	0.96	0.95	31.46	59.77	53.41
0.40	0.005	0.55	0.55	0.55	3.40	3.40	3.40
	0.01	0.61	0.61	0.61	4.92	4.96	4.94
	0.05	0.74	0.74	0.74	10.57	10.73	10.71
	0.10	0.79	0.80	0.80	13.64	14.54	14.30
	0.15	0.82	0.83	0.83	16.07	17.68	17.31
	0.20	0.84	0.86	0.86	17.23	19.50	19.08
	0.25	0.86	0.88	0.87	19.59	22.44	21.77
	0.30	0.87	0.89	0.89	19.90	25.20	23.79
	0.40	0.89	0.91	0.91	21.90	28.35	26.82
	0.50	0.90	0.93	0.93	23.63	32.49	30.30
	0.60	0.91	0.95	0.95	23.90	36.98	34.24
	0.75	0.92	0.97	0.97	24.45	46.78	42.25
	0.80	0.92	0.98	0.98	23.83	47.43	42.70
0.8	0.005	0.67	0.67	0.67	3.13	3.16	3.14
	0.01	0.72	0.72	0.72	4.56	4.59	4.57
	0.05	0.82	0.83	0.83	9.48	9.54	9.50
	0.10	0.87	0.87	0.87	12.50	13.17	13.00
	0.15	0.89	0.90	0.89	14.29	15.38	15.10
	0.20	0.90	0.91	0.91	15.78	17.61	17.12
	0.25	0.91	0.92	0.92	17.18	19.46	18.82
	0.30	0.92	0.94	0.93	17.54	21.06	20.47
	0.40	0.93	0.95	0.95	19.08	24.70	23.35
	0.50	0.94	0.96	0.96	19.87	27.96	26.15
	0.60	0.95	0.97	0.97	20.11	31.76	29.44
	0.75	0.95	0.99	0.99	20.52	40.60	37.04
	0.80	0.96	0.99	0.99	19.61	43.55	39.53
random	0.005	0.54	0.54	0.54	3.48	3.47	3.48
	0.01	0.60	0.60	0.60	4.90	4.92	4.92
	0.05	0.73	0.74	0.74	10.47	10.72	10.64
	0.10	0.79	0.79	0.79	14.16	15.13	14.85
	0.15	0.82	0.83	0.83	16.08	17.31	17.02
	0.20	0.84	0.85	0.85	18.73	20.39	19.96
	0.25	0.85	0.87	0.87	20.10	23.10	22.09
	0.30	0.86	0.88	0.88	20.77	25.09	23.89
	0.40	0.88	0.91	0.91	22.21	29.00	27.49
	0.50	0.89	0.93	0.93	23.99	34.05	31.96
	0.60	0.90	0.95	0.94	24.59	38.03	35.31
	0.75	0.91	0.97	0.97	24.95	46.61	42.27
	0.80	0.92	0.98	0.98	25.33	50.35	45.16

Table 3: Power and Stability of compared multiple testing procedures for sample size $n = 30$ in each group

ρ	π_1	Power			Stability		
		BH	Storey	Bon-EV	BH	Storey	Bon-EV
0	0.005	0.76	0.76	0.76	2.92	2.92	2.92
	0.01	0.80	0.80	0.80	4.07	4.09	4.10
	0.05	0.88	0.88	0.88	8.58	8.76	8.74
	0.10	0.91	0.91	0.91	11.01	11.52	11.38
	0.15	0.92	0.93	0.93	12.91	14.07	13.80
	0.20	0.93	0.94	0.94	14.32	15.62	15.35
	0.25	0.94	0.95	0.95	15.24	17.81	17.22
	0.30	0.94	0.96	0.95	16.14	20.07	19.53
	0.40	0.95	0.97	0.97	17.55	23.52	22.58
	0.50	0.96	0.98	0.97	18.70	27.34	25.90
	0.60	0.96	0.98	0.98	17.86	29.62	27.66
	0.75	0.97	0.99	0.99	17.91	37.12	33.60
	0.80	0.97	0.99	0.99	17.73	42.24	38.57
0.4	0.005	0.86	0.87	0.86	2.62	2.62	2.61
	0.01	0.89	0.89	0.89	3.76	3.77	3.76
	0.05	0.94	0.94	0.94	7.34	7.40	7.38
	0.10	0.96	0.96	0.96	9.18	9.95	9.81
	0.15	0.97	0.97	0.97	11.11	12.54	12.36
	0.20	0.97	0.98	0.98	11.84	13.45	13.19
	0.25	0.98	0.98	0.98	12.80	15.35	14.96
	0.30	0.98	0.98	0.98	13.50	16.98	16.48
	0.40	0.98	0.99	0.99	14.28	20.42	19.56
	0.50	0.99	0.99	0.99	14.93	23.50	22.27
	0.60	0.99	0.99	0.99	14.15	26.17	24.42
	0.75	0.99	1.00	1.00	13.06	34.86	31.92
	0.80	0.99	1.00	1.00	12.78	39.37	36.15
0.8	0.005	0.92	0.92	0.92	2.46	2.45	2.46
	0.01	0.94	0.94	0.94	3.33	3.36	3.35
	0.05	0.97	0.97	0.97	6.31	6.49	6.44
	0.10	0.98	0.98	0.98	8.10	8.83	8.74
	0.15	0.99	0.99	0.99	9.65	10.53	10.33
	0.20	0.99	0.99	0.99	10.76	12.49	12.29
	0.25	0.99	0.99	0.99	11.67	14.05	13.70
	0.30	0.99	0.99	0.99	12.14	15.42	14.90
	0.40	0.99	1.00	1.00	12.12	18.22	17.46
	0.50	0.99	1.00	1.00	12.63	21.73	20.47
	0.60	1.00	1.00	1.00	12.26	23.54	22.04
	0.75	1.00	1.00	1.00	10.94	34.44	31.65
	0.80	1.00	1.00	1.00	10.64	38.15	34.94
random	0.005	0.86	0.86	0.86	2.68	2.69	2.69
	0.01	0.89	0.89	0.89	3.73	3.75	3.75
	0.05	0.94	0.94	0.94	7.57	7.86	7.82
	0.10	0.95	0.96	0.96	9.55	10.22	10.08
	0.15	0.96	0.97	0.97	11.18	12.31	12.10
	0.20	0.97	0.97	0.97	12.09	13.40	13.14
	0.25	0.97	0.98	0.98	13.60	16.03	15.58
	0.30	0.98	0.98	0.98	13.83	16.71	16.21
	0.40	0.98	0.99	0.99	14.00	20.38	19.38
	0.50	0.98	0.99	0.99	14.42	22.65	21.48
	0.60	0.98	0.99	0.99	14.70	27.21	25.45
	0.75	0.99	1.00	1.00	14.16	35.18	32.34
	0.80	0.99	1.00	1.00	13.28	40.01	36.50

Table 4: Total number of rejections of compared multiple testing procedures for sample size $n = 5, 15, 30$ in each group

n	$m - m_0$	$\rho = 0$			$\rho = 0.4$			$\rho = 0.8$			random correlation		
		BH	Storey	Bon-EV	BH	Storey	Bon-EV	BH	Storey	Bon-EV	BH	Storey	Bon-EV
$n = 5$	50	0	0	0	0	0	0	0	0	0	0	0	0
	100	0	0	0	0	0	0	1	1	1	0	0	0
	500	1	1	1	4	4	4	16	18	16	4	4	4
	1000	4	5	4	29	37	30	113	133	118	31	41	32
	1500	12	18	12	104	142	113	289	349	314	109	146	118
	2000	33	62	36	231	321	260	515	632	571	238	326	268
	2500	77	151	90	402	567	466	782	977	885	403	566	467
	3000	151	298	184	601	865	715	1070	1360	1234	604	863	718
	4000	367	764	484	1079	1623	1349	1711	2257	2049	1071	1597	1333
	5000	674	1449	947	1626	2552	2133	2401	3280	2984	1613	2518	2109
	6000	1043	2351	1567	2227	3645	3070	3131	4429	4042	2201	3590	3026
	7500	1703	4144	2869	3199	5605	4797	4269	6369	5866	3159	5529	4726
	8000	1942	4860	3414	3537	6341	5470	4659	7097	6566	3493	6257	5389
$n = 15$	50	19	19	19	29	29	29	35	35	35	28	28	28
	100	45	45	45	64	64	64	75	76	76	63	63	63
	500	313	317	316	389	392	391	432	435	435	384	388	387
	1000	697	713	710	831	844	842	906	917	916	822	835	833
	1500	1106	1141	1135	1289	1318	1314	1389	1414	1411	1278	1306	1303
	2000	1530	1593	1583	1755	1806	1800	1877	1920	1916	1740	1792	1785
	2500	1962	2061	2045	2225	2304	2295	2367	2433	2427	2207	2287	2278
	3000	2402	2544	2523	2700	2813	2801	2859	2953	2945	2679	2794	2781
	4000	3293	3548	3513	3653	3852	3832	3841	4006	3992	3626	3829	3808
	5000	4191	4597	4545	4607	4918	4888	4820	5072	5052	4576	4895	4864
	6000	5095	5694	5622	5558	6008	5968	5795	6151	6125	5525	5981	5940
	7500	6454	7426	7325	6980	7688	7633	7244	7794	7758	6941	7663	7606
	8000	6907	8028	7921	7452	8257	8199	7724	8347	8308	7412	8237	8176
$n = 30$	50	40	40	40	46	46	46	49	49	49	45	45	45
	100	84	84	84	94	94	94	99	99	99	93	93	93
	500	461	463	463	495	497	497	511	513	512	492	494	494
	1000	949	958	957	1004	1011	1011	1028	1034	1034	1000	1007	1007
	1500	1444	1465	1463	1516	1532	1531	1545	1560	1559	1509	1527	1525
	2000	1942	1979	1976	2025	2055	2053	2059	2085	2083	2017	2047	2045
	2500	2439	2497	2492	2534	2580	2577	2572	2612	2609	2525	2571	2568
	3000	2937	3017	3011	3041	3106	3102	3082	3138	3135	3032	3098	3093
	4000	3930	4069	4059	4051	4162	4155	4096	4193	4188	4039	4153	4146
	5000	4919	5135	5120	5053	5221	5211	5100	5249	5242	5039	5211	5201
	6000	5898	6206	6186	6043	6283	6269	6093	6306	6295	6029	6274	6260
	7500	7356	7828	7800	7513	7879	7859	7566	7892	7875	7498	7873	7853
	8000	7838	8374	8342	7998	8410	8389	8052	8421	8402	7982	8407	8385
